# Palliative care for patients with motor neurone disease and their bereaved carers: a qualitative study

**DOI:** 10.1186/s12904-019-0423-8

**Published:** 2019-04-26

**Authors:** Clare Mc Veigh, Colette Donaghy, Briege Mc Laughlin, Alison Dick, Kiran Kaur, John Mc Conville, Max Watson

**Affiliations:** 1 School of Nursing and Midwifery, Queen’s University Belfast, Belfast, Northern Ireland; 2 Belfast Health and Social Care Trust, Belfast, Northern Ireland; 3 Western health and Social Care Trust, Derry, Northern Ireland; 4 School of Medicine, Dentistry and Biomedical Sciences, Queen’s University Belfast, Belfast, Northern Ireland; 5 Northern Ireland Hospice, Belfast, Northern Ireland; 6 Ulster University, Jordanstown, Northern Ireland

**Keywords:** Amyotrophic lateral sclerosis, Motor neurone disease, Palliative care, End of life, Qualitative, Bereaved carers

## Abstract

**Background:**

Internationally, it is widely accepted that holistic care is as an integral part of the care for people with motor neurone disease (MND), and their informal carers. However the optimal role of generalist and specialist palliative care, and how it integrates with specialist neurology services, is not fully established. Using a qualitative approach we sought to examine end of life care for people with MND in Northern Ireland, and the role of specialist and generalist palliative care.

**Methods:**

Qualitative study involving a convenience sample of 13 bereaved carers recruited using the Northern Ireland MND Register. Data collection consisted of semi-structured interviews with the bereaved carers of patients who had died 3–24 months previously with a diagnosis of MND. Data were analysed using thematic analysis.

**Results:**

Findings illuminated variations in relation to the levels of holistic care provided to this cohort of patients. Unmanaged respiratory and psychological symptoms caused perceived distress amongst patients. Participants’ experiences additionally highlighted reluctance amongst patients with MND to engage with services such as specialist palliative care. Conversely, for those who received input from specialist palliative care services carers portrayed these services to be of great benefit to the patient.

**Conclusions:**

Patients with MND in Northern Ireland may have many unmet holistic care needs. Key areas that require particular focus in terms of service development include neuromuscular respiratory physiotherapy and psychological services for patients. Future research must explore an optimal model of holistic care delivery for patients with MND and how this can be effectively integrated to best meet this patient cohorts palliative care needs.

**Electronic supplementary material:**

The online version of this article (10.1186/s12904-019-0423-8) contains supplementary material, which is available to authorized users.

## Background

Motor Neurone Disease (MND) is a progressive, neurodegenerative disease characterised by degeneration of the upper and lower motor neurones resulting in weakness and wasting of muscles. This typically leads to loss of mobility as well as difficulties with speech, swallowing and breathing, with survival generally between 3 and 5 years [1]. Key strategic neurological guidelines from the European Federation of Neurological Sciences (EFNS) and the National Institute for Health and Care Excellence (NICE) have recommended that all patients with MND should receive coordinated clinic-based specialist multidisciplinary care and access to specialist palliative care [2, 3]. The National Institute for Health and Care Excellence have further recommended that a member of the multi-disciplinary team should have expertise in palliative care [2]. Observational studies conducted in Ireland [4, 5], America [6] and the Netherlands [7] have provided some evidence that specialist multidisciplinary clinics positively impact upon survival and quality of life in MND, however specialist palliative care was not a core part of the multidisciplinary teams examined. For patients with MND, early referral to specialist palliative care services is recommended [3, 8]. There is some evidence to suggest that specialist palliative care improves quality of life for patients with MND and their carers [9]. Previous research has additionally illuminated that patients with MND receiving specialist palliative care in a hospice setting, were more likely to die in their preferred place of death than those not attending hospice [4]. International studies of patient preferences have found that, given adequate support, patients prefer to die at home [10].

Patients with MND in Northern Ireland (NI) are diagnosed by neurologists and attend either a general neurology clinic or a regional MND specialist clinic held within two different centres in NI. All patients, however, are referred to the regional NI MND Care Centre. There are currently six specialist inpatient facilities where specialist palliative care can be accessed by patients with MND. Local initiatives have seen the development of an End of Life strategy [11] in England in 2008, and a five year palliative and end of life care strategy called ‘Living Matters: Dying Matters’ in NI [12]. Despite these key strategic drivers, aimed at improving palliative care for patients with advanced progressive illnesses, a recent qualitative study of end of life care in MND in the United Kingdom, however, found that patients and their carers continue to experience difficulties that are not always effectively managed [13]. The present study aims to address the research question: What are the experiences of end of life care for people with MND receiving generalist and specialist palliative care in NI?

## Aim

To explore the provision of generalist and specialist palliative care in Northern Ireland, at the end of life, for people with MND from the perspective of bereaved carers.

## Methods

### Design

Due to the exploratory nature of this research it was important that the methodological approach taken aimed to explore the individual interpretations the participants associated with the phenomenon being investigated. Therefore, a broad interpretivist methodological approach was found to be the most suitable to answer the research question.. Data were collected using semi-structured interviews with bereaved carers of patients with MND.

### Settings and participants

The study recruited a total of 13 bereaved carers who cared for a patient who had died 2–24 months previously with a diagnosis of MND. There is no widely accepted time after which it is felt appropriate to approach carers about research following bereavement [14]. However it was felt that this period of time would allow for recall of the patients’ experience whilst reducing the trauma experienced by the carer. The study invited 20 bereaved carers from within the five healthcare trusts in NI who met the inclusion criteria. The deceased patients’ details were identified through the Northern Ireland MND register [15] which records patient and carer details, clinical information and involved services and therapists.. Bereaved carers had to be 18 years of age or over, English speaking, well enough to participate and having had significant involvement in the care of the patient. Eligible bereaved carers received a letter of invitation, and information about the study. If there was no response, a follow-up phone call was made after two weeks to ask if they would be willing to participate. Once a carer had agreed to take part in the study the investigator (BM) contacted the participant by phone to arrange an appropriate time for the interview. Full written and verbal informed consent was obtained prior to the interview. Thirteen bereaved carers agreed to partake in the study and participated in a semi-structured interview. Table 1 highlights the participant profiles. Participants’ ages ranged from 24 to 85 years, with a mean age of 57.3 years.

**Table 1:** Participant profiles

Carer	Gender	Relationship to patient	Living together	Patient survival from diagnosis, m	SPC^a^	Time post bereavement (months)
1	F	wife	Y	6	No	8
2	M	grandson	N	8	No	23
3	F	daughter	Y	13	No	23
4	F	wife	Y	11	No	23
5	M	husband	Y	9	No	7
6	F	daughter	Y	7	No	12
7	F	daughter	N	10	No	13
8	M	husband	Y	41	No	15
9	M	husband	Y	42	Yes – SPC Nurse/ Day hospice	18
10	F	wife	Y	36	Yes - SPC Nurse	14
11	M	husband	Y	10	Yes - Hospice (Inpatient)	18
12	F	wife	Y	100	Yes - Hospice (Inpatient)	18
13	F	wife	Y	30	Yes - SPC Nurse	24

^a^SPC, Specialist palliative care involvement

### Data collection

Data were collected through semi-structured face to face interviews conducted in the carers’ homes in 2015. Each interview was digitally recorded, transcribed verbatim and was 60–90 min in length. Researcher BM conducted the interviews with an interview schedule containing questions and prompts agreed by the research group (Additional file 1). BM was employed as a research nurse with clinical experience of MND. She also received training in qualitative interview techniques. Pseudonyms have been used for the carers to maintain confidentiality.

### Data analysis

Data were analysed by adopting King and Horrock’s approach to thematic analysis [16]. Preliminary analysis of the interview transcripts involved the development of descriptive themes to generate a list of emerging topics from the data. The next stage of analysis consisted of grouping together relevant descriptive themes to highlight the meaning of the data and illuminate interpretative themes. The final stage of analysis involved the linkage of interpretative themes to form several overarching themes. Each transcript was initially analysed by BM and then reviewed by other members of the team (CMV, MW and CD). Themes and transcripts were then discussed jointly for verification and agreement at regular meetings. The content of the transcripts was reviewed by CMV, MW, AD and CD and discussed with BM and constructive feedback given in an effort to support BM in continuing to conduct comprehensive and sensitive interviews.

### Rigor

Reflective questioning was used throughout the interviews to ensure the researcher appropriately captured the experiences of the bereaved carers, in addition to transcripts being reviewed by members of the research team. Data triangulation also occurred as the study used multiple key informants to provide a range of data sources. Rigor was enhanced through the use of thick description as detailed accounts were provided of participants’ experiences. An audit trail was also maintained to ensure an accurate account of all elements of the study were recorded.

### Ethics

Local sponsorship and governance approval was given by the Belfast Trust Research Office. Full ethical approval was successfully granted in 2013 by the Office for Research Ethics Committees Northern Ireland reference 14/NI/1034.

## Results

Three main overarching themes emerged from the data; the provision of holistic care, the biopsychosocial impact of MND, and lack of death preparedness.

### The provision of holistic care

Analysis of the bereaved carers’ interviews identified the overarching theme: *the provision of holistic care,* developed from the interpretative themes outlined below and illustrated through the carers’ quotes. Many factors contribute to good holistic care in MND, namely the availability of services both generic and specialist, timeliness of access and patient acceptance and uptake of such services. Two interpretative themes emerged;

#### Variable experiences linked to holistic service provision

Carers’ accounts conveyed variability in the level of knowledge and experience generalist healthcare professionals had in relation to the care of a patient with MND. Concern was raised about the lack of specialist knowledge of MND.

*‘That was the vibe I was getting, that MND was something she (physiotherapist) didn’t have much experience with’. (Carer 6).*

*‘It was a learning curve for a lot of the people (healthcare professionals) who helped us. I don’t think they had dealt with these situations before you know’. (Carer 13).*

However, carers who experienced specialist MND support expressed a feeling of support from the MND nurse based upon her apparent knowledge of the condition:

*‘I think she (MND nurse) was the only one who understood’. (Carer 4).*

*‘It felt good to have someone (MND nurse) to give you that wee bit more of a clear understanding as to what was going on’. (Carer 6).*

Out of the 13 patients, 5 had specialist palliative care input and carers perceived this to be of great benefit to the patient. Specialist palliative care input involved either nurse support in patients’ homes or hospice services, both inpatient and outpatient.

*‘She enjoyed her time in the hospice...they were very good to her’. (Carer 11).*

*‘I don’t know what I would have done without them (specialist palliative care sit in service) … it was great, a fantastic service. It meant you could have gone and got rest without constant interruption’. (Carer 13).*

However, some carers felt appreciative of the support provided by generalist healthcare professionals in the community care setting such as general practitioners and district nurses. Patients with MND at the end of life are often too unwell to attend hospital and so primary care input can be a valuable support.

*‘I would put it all down to the GPs (General Practitioners), the two GP’s were out nearly every day, they were fantastic’. (Carer 7).*

#### Uptake of services

There appeared also to be a lack of engagement by patients, with some often refusing services to aid their holistic needs. In some transcripts there was an indication that a lack of acceptance of the disease, as well as low mood, was contributing factors to poor engagement and lack of service uptake.

*‘Everything he (doctor) said to him he said he didn’t need it so that was just his line, “I don’t need it”….. they must have came about five or six times about the hospital bed and he sent it away ‘. (Carer 10).*

*‘He (patient) was very quiet on it...... didn’t want anybody (healthcare professionals).. I think he gave up.... he didn’t want to be bothered’. (Carer 4).*

In many transcripts a reluctance to engage with specialist palliative care services was evident. A carer indicated in one such transcript that this may have been due to lack of familiarity at an earlier stage.

*‘Daddy had totally said the hospice was a no no… maybe if they’d (specialist palliative care team) been involved a bit sooner he wouldn’t have been so negative’. (Carer 6).*

### The biopsychosocial impact of MND

Analysis of the bereaved carers’ interviews identified the overarching theme: *the biopsychosocial impact of MND*, developed from the interpretative themes outlined below and illustrated through the carers’ quotes. Patients with MND experience a wide range of symptoms with overlap between the physical and psychological effects of the disease. In addition, patients often require input from multiple healthcare professionals from different specialties due to the complexity of the disease, necessitating the need for good communication. Two interpretative themes emerged.

#### Unmanaged psychological symptomatology

Psychological symptoms were common complaints when carers were asked about the main symptoms in the last three months of life. Carers seemed to easily identify these symptoms.

*‘All night you would have heard her moaning because she didn’t want to go to sleep..... I think it was more fear than pain’. (Carer 3).*

Many carers noted an absence of psychological therapy and input within the health service. One carer expressed that psychological support would have been beneficial to the patient they cared for:

*‘Mum mentioned that she would have liked to have talked about how she was feeling spiritually... she felt counselling would have been helpful’. (Carer 5).*

#### Unmanaged respiratory symptoms and lack of recognition and anticipation

Respiratory symptoms accounted for the vast majority of physical symptoms at the end of life. In addition it is worth noting the interplay between respiratory and psychological symptoms. Concerns were raised about the recognition of respiratory impairment in patients by medical staff. Carers’ accounts illuminated an unmet need with regards to the respiratory symptoms experienced by patients with MND:

*‘He (respiratory physician) was under the impression that (husband) didn’t need any help… I kept saying he does need help….he doesn’t sleep and he feels a shortness of breath… I wish there was more knowledge about how things can escalate’. (Carer 1).*

*‘He got frightened sometimes with his breathing...... he said he couldn’t breathe and they took him in, they kept him the one night and he came back and always his breathing’. (Carer 4).*

Non-invasive ventilation (NIV) is used to alleviate the symptoms caused by the respiratory impairment in MND [3]. A lack of adjustment to NIV is likely to contribute to poor symptom control. Difficulties tolerating non-invasive ventilation were prominent, with carers reporting the most troubling symptoms as breathing, agitation and fear. There appeared to be significant overlap in patient’s respiratory and psychological symptomatology:

*‘Well the agitation and fear was solely concerned with his breathing. I mean once that machine became unattached he absolutely panicked’. (Carer 13).*

*‘We had the NIV for him but he was afraid to use it, then in the end it became kind of too strong for him anyway’. (Carer 10).*

Cough impairment is often a forerunner to ventilatory failure and presents as difficulty expectorating phlegm, a very distressing symptom for patients without appropriate management. Difficulties with phlegm expectoration were frequently described, often necessitating unplanned hospital admissions:

*‘We had the whole blue light scenario all the way to (the hospital) just for the consultant to come and apologise to us…he said I can’t do anything here... with us the big issue was the mucus and it can be frightening and people need good support…. there isn’t enough understanding of MND’. (Carer 6).*

Many carers additionally expressed that the patient they cared for struggled for breath at the end of life:

*‘It (patient’s death) was horrendous to watch… it was the gasping for breath’. (Carer 10).*

### Lack of death preparedness

Analysis of the bereaved carers’ interviews identified the overarching theme*: lack of death preparedness,* developed from the interpretative themes outlined below and illustrated through the carers’ quotes. MND is a progressive non-curative disease and guidance suggests that, where the opportunity arises, attempts should be made to discuss a patient’s wishes and make advance care plans including advance decisions to refuse treatment [2, 3]. These can be difficult and very sensitive discussions.

#### Awareness of end of life

A lack of awareness that the end of life was approaching was frequently described by carers:

*‘The thing was that we didn’t really know it was happening (patient dying) … I think it was just a real shock for them all (ward nurses)…I just don’t think they realised… it was really quick’. (Carer 13).*

Some carers perceived that the healthcare professionals involved in the patient’s care were aware the patient was approaching end of life, but this was not shared with family.

*‘She (Occupational Therapist) told us after (patient) had died that she knew she wasn’t going to last… she didn’t lie to us but she didn’t want to hurt us’. (Carer 3).*

Five patients out of 13 had made an advance decision to refuse treatment (ADRT). For those patients who had not made an ADRT a number of issues were raised including a lack of awareness of ADRT as well as lack of opportunity to discuss end of life:

*‘We never really knew anything about that (ADRT)’. (Carer 2).*

*‘It would have been good if she had had the opportunity (to discuss end of life)’. (Carer 5).*

#### Place of death

Five patients with MND died at home, one in hospice and seven in hospital. Of those who did not die at home, some carers perceived that they did not die in their preferred place:

*‘We fought with them (hospital staff) to take him home….that was his wishes.’ (Carer 2).*

Difficulties arose for some carers when patients were urgently admitted to hospital at the end of life. One carer questioned the appropriateness of medical interventions at the end of life for those that died in hospital. Another had difficulty accessing the ward to visit.

*‘They (medical team) wanted him to have more oxygen and I said no… I said I just want him to be comfortable… could we please have the oxygen that goes in the wee tubes and they said he needed more support than that…he was afraid of the masks’. (Carer 1).*

*‘I said when can we see her? And she said well visiting is not until 2pm, so we couldn’t get in.’ (Carer 5).*

## Discussion

Present findings illuminated bereaved carers’ perceptions that patients with MND often experienced variances in the holistic care delivered by healthcare professionals, with differing levels of knowledge and skills evident in relation to the illness. Concurrent with previous research [17–19], participants identified a lack of knowledge in relation to the holistic care needs and management of patients with MND amongst generalist palliative care providers. Similar to the study conducted by Bentley et al. [19], for those healthcare professionals that appeared to understand MND well, great benefits were noted by participants. However present findings additionally discovered that community healthcare professionals, such as GPs and district nurses, played a key role in managing the patients holistic care needs. Additionally, a lack of recognition and optimal management of psychological and respiratory symptoms experienced by patients with MND was evident within participant accounts. This highlights the need for a model of palliative care for patients with MND that optimises the integration between specialist MND, specialist palliative care and generalist healthcare providers [20].

Conversely, findings demonstrated perceptions that many patients with MND were reluctant to fully avail of the services offered to them. Foley et al. (2014) examined 34 patients employing qualitative interviews in an effort to assess the psycho-social processes that underpin how patients with MND engaged with healthcare services. They identified that control, reassurance, and trust are key variables that shape how people with MND engage with healthcare services [21]. If patients with MND consider the healthcare professionals involved in their care to have optimal knowledge of their condition then this may increase their engagement with services providing holistic care.

It is recognised that psychological support is an integral part of holistic MND care [3, 22]. Reported rates of depression in MND are variable with little correlation with physical disability [23], however higher levels of anxiety and depression are related to poorer health related quality of life amongst this patient cohort [24, 25]. Present findings offered perceptions that, although patients experienced various psychological symptoms, such as fear and anxiety, there was a lack of formal psychological support available. It is important that patients with MND have access to formal psychological support to optimise holistic symptom management [26]. Furthermore, there appeared to be an overlap in the respiratory and psychological symptomatology experienced by patients. Shortness of breath, fear and anxiety were often present together.

Research has demonstrated that NIV improves survival, quality of life and cognitive function [27–29] for people with MND. Present findings displayed perceptions that a lack of recognition of respiratory impairment and intervention appeared to have contributed to suboptimal symptom control. The perceptions of the bereaved carers illuminated that quite often the presentation of respiratory crisis resulted in an emergency hospital admission for the patient with MND. Additionally, experiences suggested that patients with MND had difficulties in relation to appropriately utilising NIV. These findings highlight the importance of respiratory input into MDT services as well as the need for education on neuromuscular respiratory impairment for those delivering palliative care to patients with MND. .

Our study highlighted a lack of death preparedness [30] amongst bereaved carers of patients with MND. Death preparedness can be defined as the readiness for death [31] experienced by the patient and their carer. A contributing factor to being prepared for death is also an awareness of dying [32], however carers portrayed that they were unaware the patient with MND was going to die. This finding confers with previous research demonstrating a lack of death preparedness amongst patients and carers with other forms of non-malignant conditions, such as non-malignant respiratory disease [33, 34, 19]. The present study also aligned with the theory of closed awareness which refers to the patient, and potentially the carer, being unaware of the patient’s poor prognosis however the healthcare professionals involved in their care are aware and have not shared this information [35]. This demonstrates the importance of effective information sharing between healthcare professionals, patients with MNDs and their carers. Additionally, this particular finding may provide further insight into why less than half of patients in the present study had completed an ADRT.

### Limitations

Findings only represented the perspectives of bereaved carers and not the patient’s own perspective, however caregivers can be good proxies for patients [4]. Additionally, this study did not explore the perceptions of healthcare professionals involved with this patient group which may have enhanced the findings.

## Conclusion

This study illuminated the need for the integration of neurology and palliative care when designing services for patients with MND as a key priority. This would facilitate the sharing of expertise and better support healthcare professionals to engage in advance care planning with patients with MND, and their carers. In addition a good understanding of neuromuscular respiratory impairment and its overlap with psychological symptoms at the end of life in MND is imperative for those delivering palliative care to patients. This would enable symptoms to be identified in a timely manner and effectively managed.. Finally, establishing the optimal model of integration of neurology and specialist palliative care services could serve as a template for the management of other progressive neurodegenerative disorders.

## Additional file

Additional file 1: Interview Schedule. This is the interview schedule used to guide the semi-structured interviews with bereaved carers, containing questions and prompts agreed by the research group. (DOCX 16 kb)

## Abbreviations

ADRT: Advance Decision to Refuse Treatment
GP: General Practitioner
MND: Motor Neuron Disease
NI: Northern Ireland
NIV: Non-Invasive Ventilation

## References

[1] Rowland LP, Shneider NA (2001). Amyotrophic lateral sclerosis. N Engl J Med.

[2] National Institute for Health and Care Excellence (NICE) (2016). Motor neuronee disease: assessment and management.

[3] Andersen PM, Abrahams S, Borasio GD, de Carvalho M, Chio A, Van Damme P, Hardiman O, Kollewe K, Morrison KE, Petri S (2012). EFNS guidelines on the clinical management of amyotrophic lateral sclerosis (MALS)–revised report of an EFNS task force. Eur J Neurol.

[4] Ganzini L, Johnston WS, Silveira MJ (2002). The final month of life in patients with ALS. Neurology..

[5] Traynor BJ, Alexander M, Corr B, Frost E, Hardiman O (2003). Effect of a multidisciplinary amyotrophic lateral sclerosis (ALS) clinic on ALS survival: a population based study, 1996-2000. J Neurol Neurosurg Psychiatry.

[6] Rooney J, Byrne S, Heverin M, Corr B, Elamin M, Staines A (2013). Survival analysis of irish amyotrophic lateral sclerosis patients diagnosed from 1995-2010. PLoS One.

[7] Van den Berg JP, Kalmijn S, Lindeman E, Veldink JH, de Visser M, Van der Graaff MM (2005). Multidisciplinary ALS care improves quality of life in patients with ALS. Neurology..

[8] Oliver D, Campbell C, Wright A (2007). Palliative care of patients with motor neuronee disease. Progress Palliat Care.

[9] Veronese S, Gallo G, Valle A, Cugno C, Chio A, Calvo A (2015). The palliative care needs of people severely affected by neurodegenerative disorders: a qualitative study. Progress Palliat Care.

[10] Collis E, Al-Qurainy R (2013). Care of the dying patient in the community. Bmj..

[11] Department of Health (DOH) (2008). End of life care strategy. Promoting high quality care for all adults at th end of life.

[12] Department of Health, Social Services and Public Safety Northern Ireland (DHSSPSNI) (2010). Living Matters Dying Matters. A palliative and end of life care strategy for adults in Northern Ireland.

[13] Whitehead B, O'Brien MR, Jack BA, Mitchell D (2012). Experiences of dying, death and bereavement in motor neuronee disease: a qualitative study. Palliat Med.

[14] Stroebe M, Stroebe W, Schut H (2003). Bereavement research: methodological issues and ethical concerns. Palliat Med.

[15] Donaghy C, Clarke J, Patterson C, Kee F, Hardiman O, Patterson V. The epidemiology of motor neurone disease in Northern Ireland using capture-recapture methodology. Amyotroph Lateral Scler. 11(4):374–8. 10.3109/17482960903329569 20550486

[16] King NH (2010). C. Interviews in qualitative research.

[17] Herz H, McKinnon PM, Butow PN (2006). Proof of love and other themes: a qualitative exploration of the experience of caring for people with motor neurone disease. Prog Palliat Care.

[18] O’Brien M, Whitehead B, Jack B, Douglas Mitchell J (2011). Multidisciplinary team working in motor neuronee disease: patient and family carer views. Br J Neurosci Nurs.

[19] Bentley B, O’Connor M (2016). The end of life experiences of people with motor neurone disease: family carers’ perspectives. J Palliat Med.

[20] Van Vliet LM, Gao W, DiFrancesco D, Crosby V, Wilcock A, Byrne A (2016). How integrated are neurology and palliative care services? Results of a multicentre mapping exercise. BMC Neurol.

[21] Foley G, Timonen V, Hardiman O (2014). Understanding psycho-social processes underpinning engagement with services in motor neuronee disease: a qualitative study. Palliat Med.

[22] Oliver D, Radunovic A, Allen A, McDermott C (2017). The development of the UK National Institute of health and care excellence evidence-based clinical guidelines on motor neuronee disease. Amyotroph Lateral Scler Frontotemporal Degener.

[23] McLeod JE, Clarke DM (2007). A review of psychosocial aspects of motor neuronee disease. J Neurol Sci.

[24] Van Groenestijn AC, Kruitwagen-van Reenen ET, Visser-Meily JM, van den Berg LH, Schroder CD (2016). Associations between psychological factors and health-related quality of life and global quality of life in patients with ALS: a systematic review. Health Qual Life Outcomes.

[25] Pagnini F, Marconi A, Tagliaferri A, Manzoni GM, Gatto R, Fabiani V (2017). Meditation training for people with amyotrophic lateral sclerosis: a randomized clinical trial. Eur J Neurol.

[26] Rabbitte M, Bates U, Keane M (2015). Psychological and psychotherapeutic approaches for people with motor neurone disease: a qualitative study. Amyotroph Lateral Scler Frontotemporal Degener.

[27] Lyall RA, Donaldson N, Fleming T, Wood C, Newsom-Davis I, Polkey MI (2001). A prospective study of quality of life in ALS patients treated with noninvasive ventilation. Neurology..

[28] Newsom-Davis IC, Lyall RA, Leigh PN, Moxham J, Goldstein LH (2001). The effect of non-invasive positive pressure ventilation (NIPPV) on cognitive function in amyotrophic lateral sclerosis (ALS): a prospective study. J Neurol Neurosurg Psychiatry.

[29] Bourke SC, Tomlinson M, Williams TL, Bullock RE, Shaw PJ, Gibson GJ (2006). Effects of non-invasive ventilation on survival and quality of life in patients with amyotrophic lateral sclerosis: a randomised controlled trial. Lancet Neurol.

[30] Pincombe J, Brown M, Thorne D, Ballantyne A, Mc CH (2000). Care of dying patients in the acute hospital. Progress Palliat Care.

[31] Hebert RS, Prigerson HG, Schulz R, Arnold RM (2006). Preparing caregivers for the death of a loved one: a theoretical framework and suggestions for future research. J Palliat Med.

[32] Mc Leod-Sordjan R (2014). Death preparedness: a concept analysis. J Adv Nurs.

[33] Mc Veigh C, Reid J, Larkin P, Porter S, Hudson P (2018). The experience of palliative care service provision for people with non-malignant respiratory disease and tehir family carers: an all-Ireland study. J Adv Nurs.

[34] Mc Veigh C, Reid J, Larkin P, Porter S, Hudson P (2017). The provision of generalist and specialist palliative care for patients with non-malignant respiratory disease in the north and Republic of Ireland: a qualitative study. BMC Palliat Care.

[35] Glaser BG, Strauss AL (1965). Awareness of dying.

