# Supplementary material for: Palliative care for patients with motor neurone disease and their bereaved carers: a qualitative study
Source: BMC Palliat Care. 2019 Apr 26;18:39. doi: 10.1186/s12904-019-0423-8 (PMC6486679; doi:10.1186/s12904-019-0423-8)
Supplement: Supplementary file 1 — Interview Schedule. This is the interview schedule used to guide the semi-structured interviews with bereaved carers, containing questions and prompts agreed by the research group. (DOCX 16 kb) [file 12904_2019_423_MOESM1_ESM.docx]

**Interview Schedule**

**Introduction**

Thank you for agreeing to take part in this research project. This interview should take approximately 1-1.5 hours.

Can you confirm that you have read and understood the information sheet we posted to you?

If for any reason you would like me to stop during the interview you just need to say.

We have received your consent form which you signed but would you like to confirm that you are still willing to participate in this study?

Are you aware that this interview is being recorded and will be listened to by other members of the research team?

When the interview is typed, we may send you a copy asking you to confirm that we have interpreted your answers correctly.

Having heard all of this, are you still in agreement to participate in the study and to have the interview taped?

Initially I will ask you some basic details about yourself. I will take some basic clinical information on _______________ from the NI MND Register.

Date of interview ________________________

Study Identification number **________________________**

**Profile of deceased *(will be completed from NI MND register)***

Age at death ________________________

Gender

Date of diagnosis

Disease onset (spinal, cognitive, bulbar)

Gaastrostomy? If so, when?

NIV ? If so when?

Date of death

Past medical history?

**Details of participant**

Age on last birthday

Gender

Relationship to deceased

How long did you care for **…………………………… ________________________**

On average how long per day would you have spent caring for________________ in the last 3 months before he/she died?

1-3 hours 4-6 hours more than 6 hours?

If more than 6 hours approximately how long?

1. **Symptom management**

Some people experience different problems in the last 3 months of life. Do you remember if ……………….. had any problems that stood out above all others.

**Prompt with:**

Breathlessness Spasms Pain Skin care Excess saliva Phlegm

Depression Anxiety Agitation Fear Other

How well do you think …………………………………… symptoms were managed?

**Prompt with:**

Breathlessness Spasms Excess saliva Phlegm Fear

Pain Skin care Depression Anxiety Agitation

Who were the main professionals involved in supporting you caring for/managing …………….. symptoms at the end of life?

**Prompt with:**

District nurse GP Neurologist Palliative care nurse Physiotherapist Other

Did you have confidence in the in the team who provided care at the end of …………………………. life?

**Prompt with:**

Confidence with doctors Confidence with palliative care

Confidences with nurses Other

What made you feel that way?

**Prompt with:**

Skills Training Attitude Knowledge

1. **Advanced care planning (preferred place of death/ADRT/advance statement of wishes/enduring power of attorney/ceilings of treatment)**

Do you know if ………………………………. had arranged a power of attorney after their

diagnosis?

**Prompt with:**

Legal documents

Some people make Advanced Directives. Did …………………………make an AD either written or talked about?

Was …………………………..clear about what he/she wanted or didn’t want at end of life?

Did …………………….ever discuss end of life care with you?

Do you know if he/she ever discussed end of life issues with anyone else?

**Prompt with:**

GP Nurse Consultant

Did ………………………..ever tell you where he/she wanted to spend their last days?

**Prompt with:**

Home Hospice Hospital Nursing Home

Did ……………………………die where he/she wanted to?

If not, do you know the reason he/she was not able to die where he wanted?

Did ……………………….ever get any treatment you felt was unnecessary?

**Prompt with:**

Gastrostomy IV fluids Antibiotics CPR

Do you feel that …………………………..ever needed treatment that he/she didn’t get?

**Prompt with:**

Gastrostomy IV fluids Antibiotics CPR

1. **Psychological and social support**

Aside from the medical professionals mentioned already, who else was involved in ………………………….care?

**Prompt with:**

Friends Family Priest/Minister

Local community/Neighbours Voluntary organisations

In your opinion, do you think that………………………………..felt supported during the progression of his/her illness?

If not, why not.

Did you feel supported during the progression of……………………… illness?

If not, why not?

1. **Issues around death and dying**

When………………………was dying, did you feel that he/she received the appropriate care?

**Prompt with:**

Specialist care Spiritual care Equipment Hospital transfer Medication

Were you present when……………………………..died?

As ………………………death approached, did you know that he/she was dying?

Who, if anyone, told you that …………………………was dying?

On the day/night of ………………………………….. death, did you know that death was imminent?

Who was with ……………………………………when he/she was dying?

If you decided not to be present for …………………………….death, can you tell me why?

If you were not present, what were you told about it at the time?

Who told you?

Do you know how …………………………….was in the last few hours before they died?

**Prompt with:**

Peaceful Agitated Confused Unconscious Comfortable

Do you feel that ……………………………..had a good death?

In your opinion, what made it a good death/bad death?

Overall, in relation to the time period surrounding ……..……………..death, can you tell me what was and was not helpful to you?

**Prompt with:**

Noise Lack of privacy Poor communication

Do you have any regrets about anything that happened during …………………………… end of life care and death?

Can you tell me your overriding memory of …………………….death?

In general, what was the most important aspect for you of ………………………………..actual death and dying?

**Prompt with:**

Peaceful Family present Spiritual care Other

In relation to dying with MND and for other people presently going through what you have went through, what do you think are the key issues that would help people caring for a loved one dying with MND?

If there was one final message you would like to give to those planning services for people dying with MND, what would that message be?
